# The role of sleep quality and perceived stress on depressive symptoms among tertiary hospital nurses: a cross-sectional study

**DOI:** 10.1186/s12888-023-04936-0

**Published:** 2023-06-12

**Authors:** Yi Zhou, Sha Wang, Min Liu, Gang Gan, Ning Qin, Xiaofei Luo, Chun Zhang, Jianfei Xie, Kewei Wang, Andy SK Cheng

**Affiliations:** 1 Nursing Department, The Third Xiangya Hospital, Xiangya School of Nursing, Central South University, Central South University, Changsha, Hunan China; 2 Xiangya School of Nursing, Central South University, Changsha, Hunan China; 3 Pediatrics Research Institute of Hunan Province, Hunan Children’s Hospital, Changsha, Hunan China; 4 Department of Rehabilitation Sciences, Hong Kong Polytechnic University, Hong Kong, China

**Keywords:** Depressive symptoms, Sleep quality, Mental health, Nurse practitioner

## Abstract

**Background:**

Nurses in tertiary hospitals are at high risk for depression. Understanding sleep quality and perceived stress may contribute to nurses’ mental health and health-related nursing productivity. The aim of this study was to investigate the role of sleep quality and perceived stress on depressive symptoms among nurses in tertiary hospitals.

**Methods:**

A total of 2,780 nurses (overall response rate = 91.1%) were recruited through a cross-sectional survey in 23 tertiary hospitals in China. Questionnaires included the Self-Rating Depression Scale, the Pittsburgh Sleep Quality Index, and the Chinese Perceived Stress Scale. Variables that were significant in Chi-square tests were further entered into binary logistic stepwise regression.

**Results:**

The prevalence of depressive symptoms was 60.3% (n = 1,676), of which 97.4% (n = 1,633) were female, and 77.8% were younger than 35 years (n = 1,304). Nurses who had moderate, poor, severe sleep quality and poor perceived pressure were more likely to be depressed. Master’s degree, 6–10 years of work, and physical activity were protective factors, while the opposite was the case for shift work and high dissatisfaction.

**Conclusions:**

More than half of nurses working in tertiary care hospitals reported depressive symptoms, and lower sleep quality and higher perceived stress were more associated with this. Perceived stress is an interesting concept, which may provide a new entry point for the well-known idea that there is a relationship between poor sleep quality and depression. It is possible to reduce depressive symptoms among public hospital nurses by providing information on sleep health and stress relief.

**Supplementary Information:**

The online version contains supplementary material available at 10.1186/s12888-023-04936-0.

## Highlights

The prevalence of depressive symptoms is high among tertiary hospital nurses (60.3%).

Lower sleep quality and worse perceived stress are modifiable variables relevant to depressive symptoms and may be useful for depressive symptom management strategies.

Taking job characteristics (i.e., work experience, shifts, and satisfaction) into consideration may help achieve fewer depressive symptoms in the nurse population.

**Supplementary Information:**

The online version contains supplementary material available at 10.1186/s12888-023-04936-0.

## Introduction

Depression, one of the most common mental disorders, is characterized by sadness, loss of interest/energy/confidence/self-esteem, inappropriate guilt, thoughts of death and suicide, poor concentration, and disturbed sleep and appetite [1]. Major depression is one of the non-negligible causes of global burden of diseases [2]. China’s economy has shown phenomenal growth in recent decades, however, rapid social transformation and a stressful work pace are likely to have exacerbated the increase in mood, behavioral disorders and related problems [3]. Health care in China is likewise evolving rapidly. The traditional model of health care, which focused solely on disease, has now begun to be replaced by a holistic model of care that focuses on biological, psychological, and social rehabilitation, which has led to higher demands on nursing practice. It would be irresponsible to assume that nurses are mentally healthy at all times without attempting to assess the current state of mental health. In the context of the new health care reform, nursing staff are most likely to experience severe perceived stress, anxiety, and depressive symptoms [4] and are significantly more likely than the general population [5]. Mental health, job satisfaction, health-related productivity and patient safety among nurses may be negatively impacted by depression [6, 7]. A recent qualitative study conducted in a tertiary care hospital found that nurses still held positive attitudes towards maintaining motivation at work [8]. This suggests that the nurse population may benefit more if more targeted depressive symptoms are provided and special attention to depressive symptoms is consistently maintained.

There is no strict standard definition of sleep quality. According to the current definition proposed by Professor Andrew D. Krystal, sleep quality is closely related to the Pittsburgh Sleep Quality Index score [9]. Theory suggests that there is a complex and bidirectional relationship between sleep disorders and depression. Sleep disturbance is a core secondary symptom of depression [10]. Sleep disorders also occur before depressive symptoms [11, 12]. Fang et al. proposed the idea that sleep disturbances are not only episodic to depression, but are predictive prodromes associated with ongoing or recurrent depression [13]. Improving sleep quality is an important intervention mediating pathway to alleviate depressive symptoms in nurses [14]. Furthermore, the critical role of sleep quality has been demonstrated in nursing. High levels of sleep quality have a positive effect on compassion satisfaction and a negative relationship with burnout [15]. In the low sleep quality group, impaired regulation of the HPA axis impairs nursing performance and threatens work safety [16]. This suggests that improving sleep quality may play an important role in preventing the development of depressive symptoms. In general, staff in tertiary hospitals have poorer sleep quality and more severe sleep problems, and the incidence is higher in both secondary and primary hospitals [17]. Depression dominates the sleep disorders of female nurses and has a deeper impact than other psychological disturbances [18]. It is therefore reasonable to hypothesize that sleep quality is a potential factor influencing depressive symptoms among nurses in tertiary hospitals.

Perceived stress is a psychological concept that is a key indicator of overall health status and outcomes and has attracted widespread attention among various groups of caregivers for decades [19–23]. Stress has been validated and identified as a predictor of depression in a large number of theoretical and empirical models. However, the extent to which stress exerts a negative effect depends on the individual’s perception, reaction, or evaluation of stress [24]. Simply put, the perception or evaluation of stress, rather than the objective presence of the event, determines the individual’s response to the stressful event [25]. Most success is achieved under stress; however, excessive stress can have detrimental outcomes [26]. Nurses who perceive more stress typically experience shattered self-esteem, impaired job functioning, high job stress, and severe depression [27, 28]. Perceived stress does have a unique contribution to depression [29]. The role of pathways through perceived stress is expected to play an effective role in reducing depression [30]. A flexible care management environment not only helps staff directly, but also indirectly coordinates staff efforts to manage stress and improve their own health, thereby promoting quality care [31]. High perceptions of stress are common among nurses rather than physicians or psychologists [32]. Even reporting minimal perceived stress lurks the risk of depression denial, undiagnosed depression, and even suicide [33]. These will lead to further increased public concern about nurses’ health. It is reasonable to assume that perceived stress as a key indicator of overall health may influence depressive symptoms among nurses in tertiary care hospitals.

In light of these concerns, depressive symptoms, sleep quality, and perceived stress are strategic roles to consider in nursing organizations and professions.

Improving sleep quality is important for promoting mental health [34], and reducing perceived stress has a significant effect on reducing mental health deterioration [35]. Although studies in dementia caregivers and young adults have shown that sleep quality and perceived stress contribute to depression [29, 36], and the association between poor sleep quality and depression is well known. However, considering the particular stressful concept of perceived stress and the limited resources currently available to investigate sleep quality, perceived stress and depressive symptoms among nurses in tertiary care hospitals, further research is warranted. Therefore, the aim of this study was to determine the role of sleep quality and perceived stress on depression among nurses in a tertiary care hospital in China. We propose the following hypothesis: lower sleep quality and higher perceived stress are associated with a higher likelihood of developing symptoms of depression.

## Methods

This study complies with the strengthening the reporting of observational studies in epidemiology (STROBE).

### Study design and setting

A cross-sectional study was conducted in tertiary hospitals in Hunan Province, China, from July 2020 to September 2020 using a two-stage sampling method. Tertiary hospitals, as hospitals at the county level and above, have the function of providing a high level of specialized health care services and performing a high level of teaching and research tasks. Therefore, the study sites can be considered to have homogeneity. According to the statistical bulletin on medical and health care development released by the Provincial Health and Wellness Committee in June 2019, the total number of tertiary hospitals in Hunan Province is 88. The source site of this study was all tertiary hospitals in Hunan Province, and the study sites were selected tertiary hospitals in Hunan Province. In the first stage, 23 hospitals were randomly selected from the 88 tertiary hospitals based on the proportion of tertiary class A and tertiary class B hospitals in the 14 prefecture-level administrative regions under Hunan Province. In the second stage, a random sample of nurses greater than or equal to 25% of the proportion in each selected hospital was selected. The inclusion criteria for this survey were (a) working in clinical nursing at a tertiary hospital; (b) working for at least 1 year; and (c) voluntary participation. Nurses who were not working due to annual leave, maternity leave, sick leave, etc. were not included.

### Data collection procedure

First, the project leader contacted the administrators of the selected hospitals and the head nurses of the selected wards through the Provincial Health Council and explained the purpose of the study in order to gain their support. Secondly, the head nurses placed envelopes filled with questionnaires at the nurses’ stations. The selected nurses in each ward were encouraged to participate in this study and fill out the questionnaires. Each questionnaire took 15–20 min to complete. Third, nurses participating in the study were asked to place the questionnaires back into the envelopes and seal them. Once completed, the researcher was responsible for collecting the envelopes.

### Measurements

The questionnaire consisted of the following components: an exposure variable of primary interest for this study, two outcome variables, and several confounding variables.

Exposure: The Self-Rating Depression Scale (SDS) was designed by Zung in 1965 and contains 20 items [37]. Each item has a score ranging from *none or very little* (1) to *most or all of the time* (4), indicating the frequency with which the subject has experienced depressive symptoms in the past seven days. The raw score is the sum of all 20 items and then multiplied by 1.25 to obtain the standardized score. A total score > = 53 indicates the presence of depression in the subject [38, 39]. The higher the score, the more severe the depressive symptoms. The scale’s Cronbach’s alpha value was 0.892 in the previous study [40] and 0.860 in the present study, both showing high reliability.

Outcome 1: The Pittsburgh Sleep Quality Index (PSQI) is the most commonly used self-administered questionnaire to assess quality of sleep in both clinical and research settings. The PSQI consists of 19 items in seven dimensions: sleep quality, sleep duration, sleep latency, sleep disturbances, habitual sleep efficiency, use of sleeping medications and daytime dysfunction. Each item is scored between *no difficulty* (0) and *severe difficulty* (3), with a total score ranging from 0 to 21. Higher total scores indicate poorer sleep quality. A total score < = 5 is considered to have good sleep quality; a total score of 6–10 is indicative of moderate sleep quality, 11–15 indicates poor sleep quality, and above 16 indicates severe sleep quality. The Chinese version of the PSQI has been validated with a Cronbach’s alpha of 0.697 and 0.790 among Chinese nurses [41]. In the present study, Cronbach’s alpha was 0.900.

Outcome 2: The Perceived Stress Scale (PSS), developed by Cohen [42], is the most classic tool for assessing stress perception. The Chinese version, revised by Yang [43], consists of 14 individual items and yield two dimensions: tension, and sense of being unable to control stress. A 5-point Likert scale was used, with options ranging from *never* (0) to *very often* (5). Scores on PSS range from 0 to 56, with higher scores representing greater perceived stress. The Cronbach’s alpha for nurses in Chinese general teaching hospitals was 0.820 [44] and 0.901 for nurses in tertiary hospitals in this study.

Confounding variables: Based on past literature, several sociodemographic characteristics are risk factors for depressive symptoms: increasing age, lower level of education, shorter duration of employment, lower position, lower job satisfaction [45], frequent night shifts [46], and infrequent exercise [47]. Thus, demographic characteristics included gender, age, education, working experience (in years), position, shift work, work satisfaction and physical activity. The specific measurement questions for confounding variables can be found in the Supplementary File.

### Ethical considerations

The co-authors guarantee the legitimacy and soundness of this study and ensure compliance with all the principles of the Declaration of Helsinki. This study was approved by the Institutional Review Board (IRB) of the Third Xiangya Hospital, Central South University (Approval no. 2017-S559). Each nurse participating in the study provided informed consent prior to completing the required data collection forms.

### Data analysis

The collected data were entered into an Excel spreadsheet and the survey responses of 2,780 participants were analyzed. IBM SPSS Statistics for Windows version 25.0 Statistical analysis included descriptive statistics to examine frequencies and percentages, and inferential statistics to examine associations among variables. Box plots were used to analyze the distribution of data from PSQI and PSS. Chi-square analyses were performed to assess the associations between categorical variables and depressive symptoms. Variables that are significant in Chi-square tests will be further entered into binary logistic stepwise regression. After adjustments for confounding variables, binary logistic stepwise regression analyses showed the relationship between sleep quality, perceived stress, and depressive symptoms.

Confusion matrices were provided to show the classification accuracy of the three logistic regression models. The parameters ø and *V* of the effect size of the chi-square test were also provided, considering that the effect size decreases with increasing sample size [48]. The predetermined alpha level was 0.05 [49].

## Results

Descriptive characteristics are presented in Table 1. A sample of 3,050 nurses agreed to participate in the survey, and 2,780 nurses fully completed. The overall response rate was 91.1%. Most of the participants were female nurses (97.7%); 26–35 years of age (26–30 years = 26.0%, 31–35 = 34.3%); had a bachelor’s degree (82.9%); had worked for less than 15 years (≤ 5 years = 20.7%; 6–10 years = 33.6%; 11–15 years = 25.8%); and held the position of primary nurse (45.1%) or senior nurse (44.9%). Nearly 80% of the participants worked night shifts and 53.8% were dissatisfied with their jobs (neutral = 38.0%; dissatisfied = 13.2%; very dissatisfied = 2.6%). Three-fifths of the subjects participated in physical activity (61.5%).

**Table 1:** Demographic characteristics of nurses with depressive symptoms. (N = 2,780)

Variables	All participants n (%)	Depressive symptoms n (%)	ø/*V*	*χ* ^*2*^	*p*-value
**Gender**			-0.025	1.709	0.191
Women	2717 (97.7)	1633 (97.4)			
Men	63 (2.3)	43 (2.6)			
**Age (in years)**			0.129	46.592	< 0.001
≤ 25	408 (14.7)	256 (15.3)			
26–30	724 (26.0)	440 (26.2)			
31–35	955 (34.3)	608 (36.3)			
36–40	397 (14.3)	246 (14.7)			
41–45	133 (4.8)	62 (3.7)			
> 45	163 (5.9)	64 (3.8)			
**Education**			0.070	13.675	0.003
Diploma	356 (12.8)	217 (13.0)			
Bachelor	2304 (82.9)	1405 (83.8)			
Master	112 (4.0)	52 (3.1)			
Doctorate	8 (0.3)	2 (0.1)			
**Working experience (in years)**			0.150	62.427	< 0.001
≤ 5	575 (20.7)	369 (22.0)			
6–10	934 (33.6)	551 (32.9)			
11–15	716 (25.8)	479 (28.6)			
16–20	251 (9.0)	151 (9.0)			
> 20	304 (10.9)	126 (7.5)			
**Position**			0.112	35.108	< 0.001
Nurse	60 (2.2)	47 (2.8)			
Primary nurse	1255 (45.1)	786 (46.9)			
Senior nurse	1249 (44.9)	748 (44.6)			
Vice professor	216 (7.8)	95 (5.7)			
**Shift work**			0.197	107.427	< 0.001
None	489 (17.6)	193 (11.5)			
Yes	2291 (82.4)	1483 (88.5)			
**Work satisfaction**			0.284	224.973	< 0.001
Very satisfied	104 (3.7)	36 (2.1)			
Satisfied	1180 (42.5)	553 (33.0)			
Neutral	1057 (38.0)	741 (44.2)			
Dissatisfied	366 (13.2)	283 (16.9)			
Very dissatisfied	73 (2.6)	63 (3.8)			
**Physical activity**			-0.146	59.147	< 0.001
None	1069 (38.5)	741 (44.2)			
Yes	1711 (61.5)	935 (55.8)			
**Sleep quality**			0.467	606.414	< 0.001
Good	613 (22.1)	189 (11.3)			
Moderate	1337 (48.1)	719 (42.9)			
Poor	712 (25.6)	661 (39.4)			
Severe	118 (4.2)	107 (6.4)			
**Perceived stress**			0.256	182.369	< 0.001
Normal	1579 (56.8)	780 (46.5)			
Poor	1164 (41.9)	865 (51.7)			
Severe	37 (1.3)	31 (1.8)			

Note

The *V*-coefficient is a correlation coefficient based on the chi-square statistic and is generally used for columnar tables where both the number of rows and columns are greater than 2. The ø correlation coefficient is also a correlation coefficient based on the chi-square statistic, and it is applied when at least one of the two categories is a dichotomous variable. Both *V* and ø are reference indicators of effect size

**Fig. 1:**
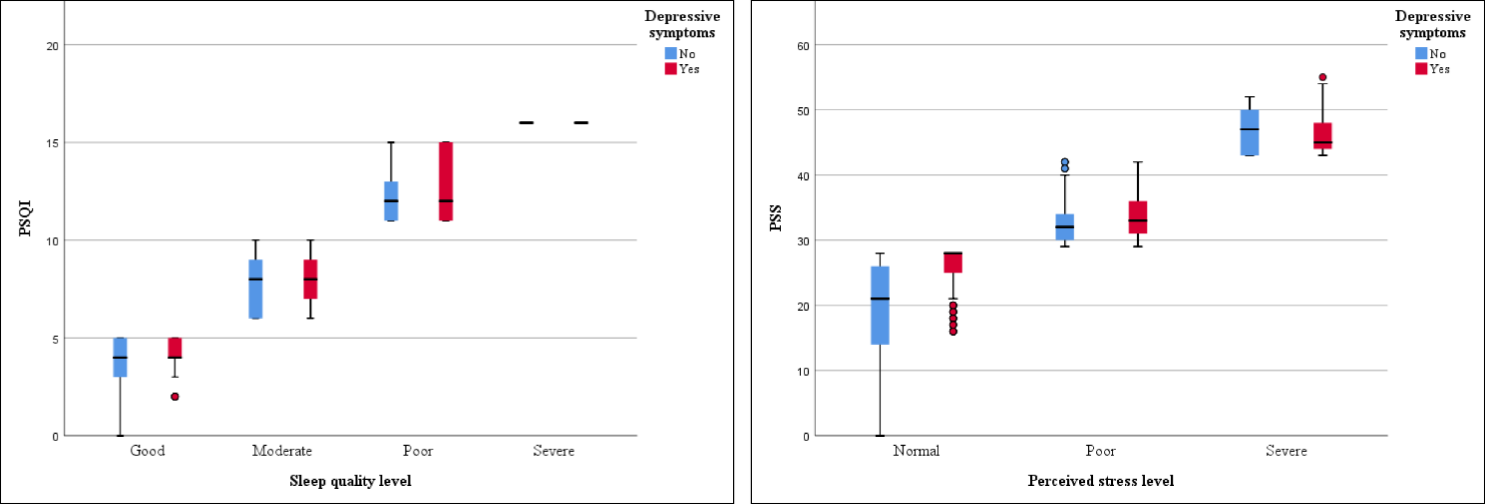
Box plot of PSQI and PSS by presence or absence of depressive symptoms. Note: PSQI = Pittsburgh Sleep Quality Index; PSS = Perceived Stress Scale

The prevalence of depressive symptoms among the participants was 60.3%. Only 22.1% reported good sleep quality, 48.1% moderate sleep quality, and 29.8% poor to severe sleep quality. While 56.8% did not report perceived stress, 41.9% had poor perceived stress, and 1.3% reported severe perceived stress. Participants with symptoms of depression were more likely to have poor sleep quality and more perceived stress (Table 1). Depressed nurses had significantly higher PSQI and PSS scores than nurses without depressive symptoms, except for the severe rating on the PSS (Fig. 1).

After full adjustment for socioeconomic status, the associations of sleep quality, perceived stress and the depressive symptoms were reduced, but still significant. In the further adjusted model (Model 3, Table 2), nurses with a master’s degree (odds ratio (OR) [95% confidence interval (95% CI)]: 0.493 [0.280–0.868]) appeared to be less likely to experience depressive symptoms than those with a diploma degree. Nurses who had worked for 6–10 years (0.649 [0.427–0.985]) appeared to be less likely to experience depressive symptoms than those with ≤ 5 years. Age and position were not associated with depressive symptoms of participants. Nurses who expressed neutral (2.330 [1.437–3.777]), dissatisfied (2.659 [1.553–4.553]), and very dissatisfied (2.517 [1.022–6.198]) with their jobs were more likely to have depressive symptoms compared to nurses who felt very satisfied. Shift work (1.926 [1.474–2.517]) were found to increase the chance of depressive symptoms, while engagement in physical activity (0.720 [0.594–0.872]) was found to have a protective effect (*p* < 0.001). As sleep quality deteriorated, participants were progressively more likely to face depressive symptoms: moderate (2.066 [1.661–2.568]), poor (19.584 [13.770-27.854]), and severe (14.285 [7.296–27.966]). Poor perceived stress (2.061 [1.703–2.494]) was also a factor that increased the odds of depressive symptoms (*p* < 0.001). Model 3 showed that 37.8% of the variance in depressive symptoms was explained, with a classification accuracy of 73.5%, details of which can be seen in Table 3.

**Table 2:** Binary logistic stepwise regression

Variables	Model 1 OR (95% CI)	Model 2 OR (95% CI)	Model 3 OR (95% CI)
**Sleep quality**			
Good	1	1	1
Moderate	2.610 (2.132–3.195)***	2.340 (1.904–2.876)***	2.066 (1.661–2.568)***
Poor	29.076 (20.852–40.543)***	24.786 (17.638–34.830)***	19.584 (13.770-27.854)***
Severe	21.822 (11.463–41.544) ***	17.648 (9.138–34.082)***	14.285 (7.296–27.966)***
**Perceived stress**			
Normal		1	1
Poor		2.109 (1.756–2.532) ***	2.061 (1.703–2.494)***
Severe		0.835 (0.319–2.185)	0.571 (0.215–1.514)
**Age**			
≤ 25			1
26–30			1.126 (0.722–1.754)
31–35			1.341 (0.793–2.267)
36–40			1.440 (0.746–2.778)
41–45			0.943 (0.378–2.353)
> 45			1.023 (0.394–2.655)
**Education**			
Diploma			1
Bachelor			0.811 (0.589–1.117)
Master			0.493 (0.280–0.868)*
Doctorate			0.714 (0.096–5.312)
**Working experience**			
≤ 5			1
6–10			0.649 (0.427–0.985)*
11–15			0.942 (0.564–1.572)
16–20			0.901 (0.458–1.770)
> 20			0.673 (0.279–1.626)
**Position**			
Nurse			1
Primary nurse			1.076 (0.485–2.390)
Senior nurse			1.116 (0.488–2.548)
Vice professor			1.482 (0.581–3.782)
**Shift work**			
None			1
Yes			1.926 (1.474–2.517)***
**Work satisfaction**			
Very satisfied			1
Satisfied			1.226 (0.763–1.970)
Neutral			2.330 (1.437–3.777)***
Dissatisfied			2.659 (1.553–4.553)***
Very dissatisfied			2.517 (1.022–6.198)*
**Physical activity**			
None			1
Yes			0.720 (0.594–0.872)***
**Model diagnosis and fit**			
Nagelkerke *R*^*2*^	0.298	0.323	0.378
*p*-value	1.000	0.011	0.966

Note

Model 1: adjusted for sleep quality; Model 2: adjusted for sleep quality and perceived stress; Model 3: adjusted for sleep quality, perceived stress, age, education, working experience, position, shift work, work satisfaction, and physical activity. CI = Confidence Interval; OR = Odds Ratio; ***p < 0.001, **p < 0.01, *p < 0.05

**Table 3:** Confusion matrix of binary logistic stepwise regression

Model		Predicted count		Accuracy %
	Actual count	Depressive symptoms	No depressive symptoms	
1	Depressive symptoms	1487	189	68.7
	No depressive symptoms	680	424	
2	Depressive symptoms	1114	562	70.3
	No depressive symptoms	264	840	
3	Depressive symptoms	1324	352	73.5
	No depressive symptoms	385	719	

## Discussion

This cross-sectional survey study explored the association between nurses’ sleep quality, perceived stress, job characteristics, and depressive symptoms. The poorer the quality of sleep, the greater the perceived stress, the lower the job satisfaction, the need to work shifts, and the greater the odds of depressive symptoms. A significant portion of nurses had symptoms of depression (60.3%), similar to a study in northwest China [45], suggesting that depressive symptoms among Chinese nurses are currently more severe than thought.

The estimated prevalence of high depressive symptoms may be related to many of the following factors. We included only nurses in tertiary hospitals. As part of the Chinese health sector, tertiary hospitals have major responsibilities for high-quality nursing care, research projects and nursing degree education programs [50]. The ensuing stress of preparing for and performing independent nursing tasks may negatively affect nurses’ health. Although the adjusted model did not show a significant effect of gender and age on depressive symptoms, characteristics such as young female nurses have been shown to be associated with an increased risk of depression [51–54]. In particular, about two-thirds of participates are in young adulthood (aged 20–35), and this period is well known to be strongly connected with later health and key point to overall health status [55, 56]. When depressive symptoms are displayed during this critical period, they must be taken seriously. Moreover, the adjusted model showed that those with a master degree had significantly fewer depressive symptoms than those with a diploma degree, which supports the results of Chi-square test analysis. This is similar to recent finding that cardiac nurses with lower academic qualification (bachelor versus master) had higher risk of depression [57]. The reason may be that the professionalism and psychological diathesis are more typically higher among nurses who receive more education [58]. However, it is worth noting that having a doctoral degree did not show a significant reduction in the odds of depressive symptoms, and the underlying differences may need to be explored in more studies. Therefore, depressive symptoms in young female nurses with lower-level degrees should be a cause for concern.

In the present study, shift work was associated with an increase in depressive symptoms. Taylor et al. suggested that individuals who work shifts are more likely to suffer from depression, particularly those with shift work disorder (SWD). This serious disorder is not only concern to nurses’ health (more severe depression), but is also closely related to patient safety (near-miss accident/error) [46]. An integrative review including 37 studies suggested that the association between shift work and psychological outcomes of nurses highly depends on contextual and personal factors, such as hours of sleep, morningness and eveningness, and leave entitlement [59]. Of particular interest is the fact that chronotype influences the risk of depression, regardless of whether or not rotating night shift [60]. These could inform nursing managers that improving quality of care is significantly associated with high negative mindset and negative psychology among nurses. In the present study, greater job dissatisfaction was associated with an odd increase in depressive symptoms. Previous studies have reported that job satisfaction is a significant predictor of depressive symptoms [61, 62]. On the other hand, working for 6 to 10 years seems to be less likely to be associated with depressive symptoms than working for less than 5 years, somewhat similar to a recent study in Pakistan that reported that working less than 5 years was associated with depression [52]. In any case, hospitals play a key role in considering job characteristics (i.e., work shifts, satisfaction, and tenure) to protect nurses from depression.

After adjusting for potential confounding variables, in the present study, poorer sleep quality was associated with increased odds of depressive. In terms of physiology, potential biological pathways in the development of sleep disorders and depressive symptoms t are commonly considered as HPA axis dysregulation [63]. Moreover, a Norwegian longitudinal study indicated that insomnia was directly associated with future depressive symptoms [64]. This is mainly due to the physiological incongruity of circadian rhythms and shift work, a common working environment in general hospitals that may contribute to sleep quality and vulnerability to depression [65]. Previous study have also found that nurses with depressive comorbidities are more likely to report sleep disorders than nurses with other types of comorbidities, such as anxiety and GI disorders) [66], especially during the months from the winter solstice to the vernal equinox. The study found that depressive symptoms showed a small but significant peak statistic during this time [67]. Specifically, nurses with comorbid depressive symptom distress sought more sleep disorder visits in the winter than in the summer [68]. Possible reasons for the seasonal patterns of sleep disorder are as follows: nurses with comorbid depression may be influenced by lower sunshine hours; and this crowd may become more sensitive to weather variations during the winter or spring. Since our findings found an association between poor sleep quality and depression during certain summer months, it is reasonable to assume that there may be a stronger association between sleep quality and depression during winter or spring, which warrants more attention in the future. Thus, a supportive environment characterized by circadian phenotypes and seasonal differences is important to help improve sleep quality and may help reduce depressive symptoms.

In the full model, perceived stress was also found to significantly influence depressive symptoms in nurses. In a longitudinal survival analysis with 33,060 cases, increased perceived stress and depressive symptoms were associated with the experience of leaving the profession [69]. It is worth emphasizing that the nurses in this study worked in tertiary care hospitals, a group that reports little optimistic data on perceived stress [70], a phenomenon that likely contributes to high turnover rates and further strains on nursing human resources. In our study, physical activity, an important factor influencing depressive symptoms, has been shown to be an important part of personal resources for coping with stress [71], and in particular, the ability to exercise consciously may be key to improving the effects of perceived stress on depression [22], and hospitals should pay more attention to helping nurses follow a healthy lifestyle. More importantly, organizational efforts focused on promoting nurses to take responsibility for their own health (physical and mental) are urgently needed with the aim of effectively improving the quality of nursing practice and patient outcomes [72]. Furthermore, an American study of university nursing students showed that perceived stress mediated the relationship between sleep quality and depressive symptoms [73], which emphasizes not only sleep promotion but also the identity of stress perception for improving mental health.

It is important for managers to relieve nurses’ depressive symptoms, which should not be considered just a representative indicator, but another step toward achieving quality care and ensuring patient safety. When nurses are at risk for depressive symptoms, it is important to actively consider improving sleep quality and reducing perceived stress. Hospitals also have a key role in considering job characteristics (i.e., shift work, job satisfaction) to protect their nurses from developing depressive symptoms. We recommend that content related to sleep hygiene be incorporated into routine nursing education. In addition, effective stress regulation strategies should be implemented to enable nursing staff to increase their resilience to perceptions.

The present study has some limitations. First, due to the cross-sectional nature of the data, no causal relationship could be found for these variables. Second, as of the end of 2019, the total number of registered nurses in China was about 4.45 million [74], with male nurses accounting for approximately 3% and female nurses accounting for approximately 97%. Therefore, the sample for this study was heavily skewed towards female participants. Third, in this study, the minimum number of years of work for participants was set at 5 years. A longitudinal study found that sleep quality, perceived stress, and depressive symptoms worsened and then improved during the first two years of employment among nurses, reminding us that this information may have been missed in our study [75]. However, there are outstanding strengths. Large-scale studies on nurses from different hospitals may help to generalize the results. Thus, by studying a homogeneous group of nurses, it would make sense to conduct studies that can provide customized solutions.

## Conclusion

The prevalence of depressive symptoms among nurses in Chinese tertiary care hospitals was 60.3%. The current study provides new insights that lower sleep quality and poorer perceived stress are more associated with depressive symptoms, especially if nurses have more shift work and less work experience or satisfaction. Therefore, considering the critical role of sleep quality and perceived stress, it is crucial to minimize depressive symptoms among nurses in public hospitals. In addition, creating a supportive environment by providing adequate job resources ensures a positive perception of healthcare work and a willingness to stay in the organization.

## Electronic supplementary material

Below is the link to the electronic supplementary material.

Supplementary Material 1

## Data Availability

The datasets generated and/or analyzed in this study are available upon reasonable request to the corresponding authors.
