# Supplementary material for: The role of sleep quality and perceived stress on depressive symptoms among tertiary hospital nurses: a cross-sectional study
Source: BMC Psychiatry. 2023 Jun 12;23:416. doi: 10.1186/s12888-023-04936-0 (PMC10258928; doi:10.1186/s12888-023-04936-0)
Supplement: Supplementary file 1 — Supplementary Material 1 [file 12888_2023_4936_MOESM1_ESM.docx]

**Questionnaire 1: General demographic information**

1. Gender: □Male □Female
2. Age: □≤25 years □26~30 years □31~35 years 36~40 years □>40 years
3. Highest education: □Secondary school □College □Bachelor's degree □Master's degree and above
4. Length of service: □≤5 years □6~10 years □11~15 years □16-20 years □>20 years
5. Technical title: □Nurse □Nurse practitioner □Nurse practitioner in charge □Deputy chief nurse and above
6. Night shift: □Yes □No
7. Job satisfaction: □Very satisfied □Satisfied □Uncertain □Unsatisfied □Very dissatisfied
8. Physical exercise: □Yes □No

**Questionnaire 2: Self-Rating Depression Scale (SDS)**

Please choose according to your own feelings:

A: None or very little of the time (no more than one day in the past week);

B: A small amount of time (1-2 days in the past week);

C: A significant amount of time (3-4 days in the past week);

D: Most or all of the time (5-7 days in the past week);

| Item | A | B | C | D |
| --- | --- | --- | --- | --- |
| 1. I feel sullen and depressed |  |  |  |  |
| 2. I feel that the morning is the best time of the day |  |  |  |  |
| 3. I cry or feel like crying in bursts |  |  |  |  |
| 4. I don't sleep well at night |  |  |  |  |
| 5. I eat as much as usual |  |  |  |  |
| 6. I feel as happy as ever when I am in close contact with the opposite sex |  |  |  |  |
| 7. I find that I am losing weight |  |  |  |  |
| 8. I suffer from constipation |  |  |  |  |
| 9. My heart beats faster than usual |  |  |  |  |
| 10. I feel tired for no reason |  |  |  |  |
| 11. My mind is as clear as usual |  |  |  |  |
| 12. I don't find it difficult to do things that I often do |  |  |  |  |
| 13. I feel restless and can't calm down |  |  |  |  |
| 14. I have hope for the future |  |  |  |  |
| 15. I get angry and excited more easily than usual |  |  |  |  |
| 16. I feel that it is easy to make decisions |  |  |  |  |
| 17. I feel that I am a useful person and that someone needs me |  |  |  |  |
| 18. I have had an interesting life |  |  |  |  |
| 19. I think others would have a better life if I were dead |  |  |  |  |
| 20. I am still interested in the things that normally interest me |  |  |  |  |

The scale consists of 20 items, each with 20 positive or negative scores, on a 4-point Likert scale. Choice A is assigned 1 point, choice B is assigned 2 points, choice C is assigned 3 points, and choice D is assigned 4 points.The sum of the scores obtained from all entries is the total rough score, and the total rough score is multiplied by 1.25 and rounded to the nearest whole number for the standard score. A standard score greater than or equal to 53 indicates the presence of depression. The higher the standard score, the more severe the depression.

**Questionnaire 3: The Pittsburgh Sleep Quality Index (PSQI)**

Please answer the following questions according to your actual situation in the past month:

1. In the past month, you usually went to bed at __ o'clock at night;

2. In the past month, it usually takes __ minutes to fall asleep each night;

3. In the past month, you usually get up at ____ in the morning;

4. In the past 1 month, you usually sleep ____ hours per night (not equal to the time spent in bed)

Please use " " to mark the most appropriate answer to the following questions:

5. In the past month, you have been troubled by the following conditions that affect your sleep:

a. Difficulty in falling asleep (cannot fall asleep within 30 minutes) ①No ②<1 time/week ③1~2 times/week ④≥3 times/week;

b. Waking up easily or early at night ①No ②<1 time/week ③1~2 times/week ④≥3 times/week;

c. Going to the toilet at night ①No ②<1 time/week ③1~2 times/week ④≥3 times/week:

d. Poor breathing ①No ②<1 time/week ③1~2 times/week ④≥3 times/week;

e. High cough or snoring ①No ②<l times/week ③1~2 times/week ④≥3 times/week;

f. Feeling cold ①No ②<l times/week ③1~2 times/week ④≥3 times/week;

g. Feeling hot ①No ②<1 time/week ③1~2 times/week ④≥3 times/week;

h. Nightmares ①No ②<1 time/week ③1~2 times/week ④≥3 times/week;

i. Pain and discomfort ①No ②<1 time/week ③1~2 times/week ④≥3 times/week;

j.Other things that affect sleep ①No ②<l/week ③1~2 times/week ④≥3 times/week;

If yes, please explain;

6. In the past month, in general, you think your sleep is very good ① very good ② better ③ poor ④ very poor;

7. In the past month, you have used medication to induce sleep ①No ② <1 time/week ③1~2 times/week ④≥3 times/week;

8. In the past month, do you often feel sleepy ①No ②<1 time/week ③1~2 times/week ④≥3 times/week;

9.In the past month, do you have low energy to do things ①No ②Sometimes ③Sometimes ④Frequently;

10. Have you had any of the following in the past month (ask your bedmate):

a. Snoring loudly ①No ②<1 time/week ③1~2 times/week ④≥3 times/week;

b. Do you have long pauses in breathing (breath-holding) during sleep? ①No ②<1 time/week ③1~2 times/week ④≥3 times/week;

c. Do you have to kick or move your legs during sleep due to leg discomfort? ①No ②<1 time/week ③1~2 times/week ④≥3 times/week;

d. During sleep, do you turn or fall asleep? ①No ②<1 time/week ③1~2 times/week ④≥3 times/week;

e. Do you have any other special conditions during sleep ①No ②<l times/week ③1~2 times/week ④≥3 times/week;

The quality of sleep in the last month was evaluated by 19 self-assessment items, of which the 19th self-assessment item was not involved in the scoring. The 18 self-assessment items could be combined into 7 components (sleep quality, sleep smell, sleep duration, sleep efficiency, sleep disorder, hypnotic medication, and daytime function). Each component is scored on a scale of 0-3, and the cumulative score of each component is the total PSQl score, which ranges from 0 to 21.

**Questionnaire 4: Perceived Stress Scale (PSS)**

Please recall how often each of the following situations has occurred in the last month.

| Item | Never | Occasionally | Sometimes | Often | Always |
| --- | --- | --- | --- | --- | --- |
| 1. Feeling upset when something unpredictable happens |  |  |  |  |  |
| 2. Feeling unable to control the important things in your life |  |  |  |  |  |
| 3. Feeling nervous and stressed |  |  |  |  |  |
| 4. Successfully deal with annoying life problems |  |  |  |  |  |
| 5. Feeling that you are effectively handling important changes in your life |  |  |  |  |  |
| 6. Feeling confident in the ability to deal with my own personal problems |  |  |  |  |  |
| 7. Feel that things are going well |  |  |  |  |  |
| 8. Find themselves unable to handle all the things they have to do |  |  |  |  |  |
| 9. Have a way to control the annoying things in life |  |  |  |  |  |
| 10. Often feel that I am the master of things |  |  |  |  |  |
| 11. Often get angry because many things happen that are beyond my control |  |  |  |  |  |
| 12. Often think of some things they must accomplish |  |  |  |  |  |
| 13. often able to master the time schedule |  |  |  |  |  |
| 14. often feel that difficult things are piling up and I can't overcome them |  |  |  |  |  |

The questionnaire consisted of 14 items categorized into 2 subscales: tension (7 items) and sense of being unable to control stress (7 items). Items 4, 5, 6, 7, 9, 10, and 13 were positively described items, belonging to the sense of being unable to control stress dimension, and were scored in the reverse direction; items 1, 2, 3, 8, 11, 12, and 14 were negatively described items, belonging to the sense of tension dimension, and were scored in the positive direction. The total scores ranged from 0 to 56, and the higher the score, the greater the psychological stress. The 0-28 of scores denote normal of the stress, 29-42 of scores denote a little bit of the stress reminding yourself to pay attention to the stress decompression, and 43-56 of scores denote higher the stress needing for external resources for the stress decompression.
